# Supplementary material for: DMRforPairs: identifying Differentially Methylated Regions between unique samples using array based methylation profiles
Source: BMC Bioinformatics. 2014 May 15;15:141. doi: 10.1186/1471-2105-15-141 (PMC4046028; doi:10.1186/1471-2105-15-141)
Supplement: Additional file 2 — DMRforPairs output for the comparison of A431-MCF7 and NA17018-NA17105. Please start from the HTML files in each folder. Available via the BMC Bioinformatics website. [file 1471-2105-15-141-S2.zip › 1394847754114233_MOESM2_ESM/A431_MCF7/figures/27796.pdf]

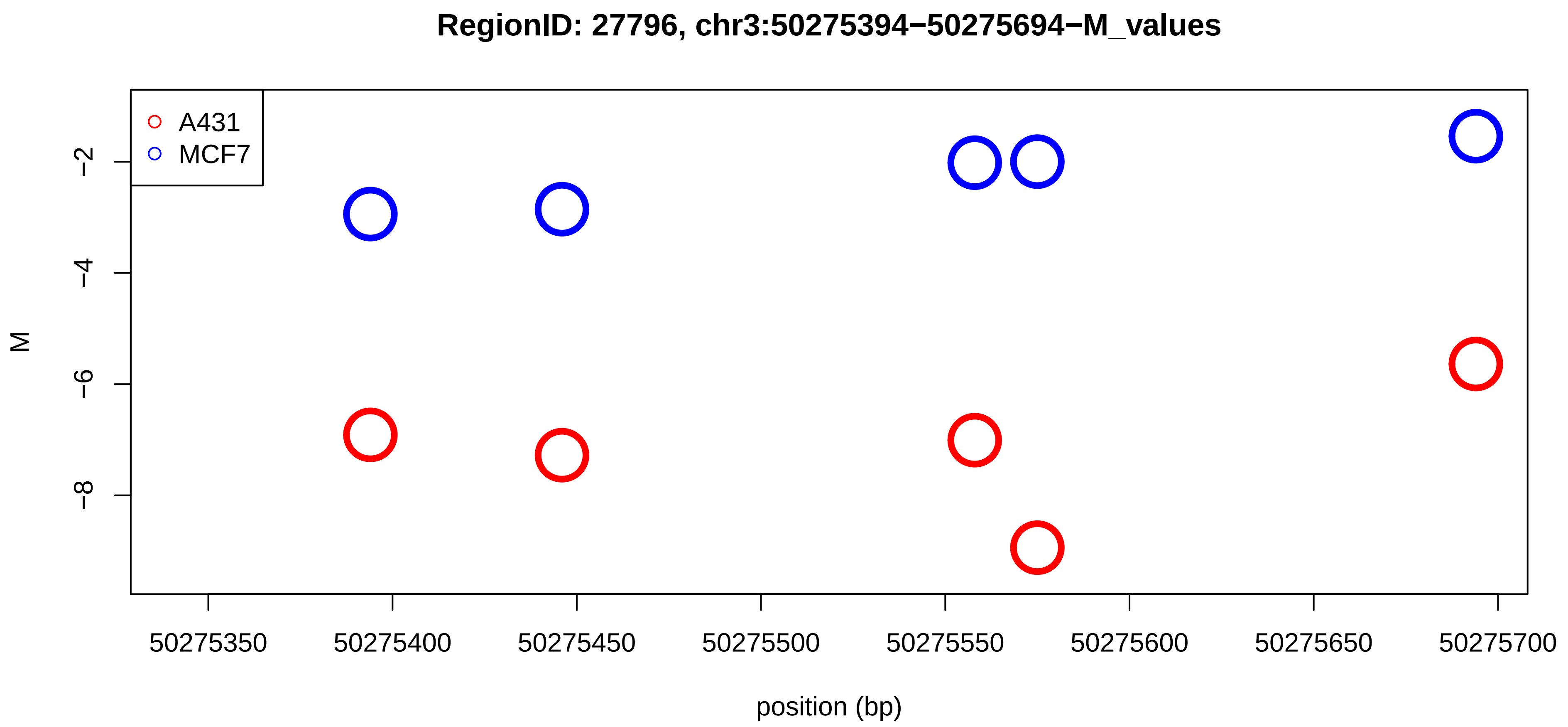
RegionID: 27796, chr3:50275394−50275694−M_values
A431
MCF7
M
−2
−4
−6
−8
50275350
50275400
50275450
50275500
50275550
50275600
50275650
50275700
position (bp)

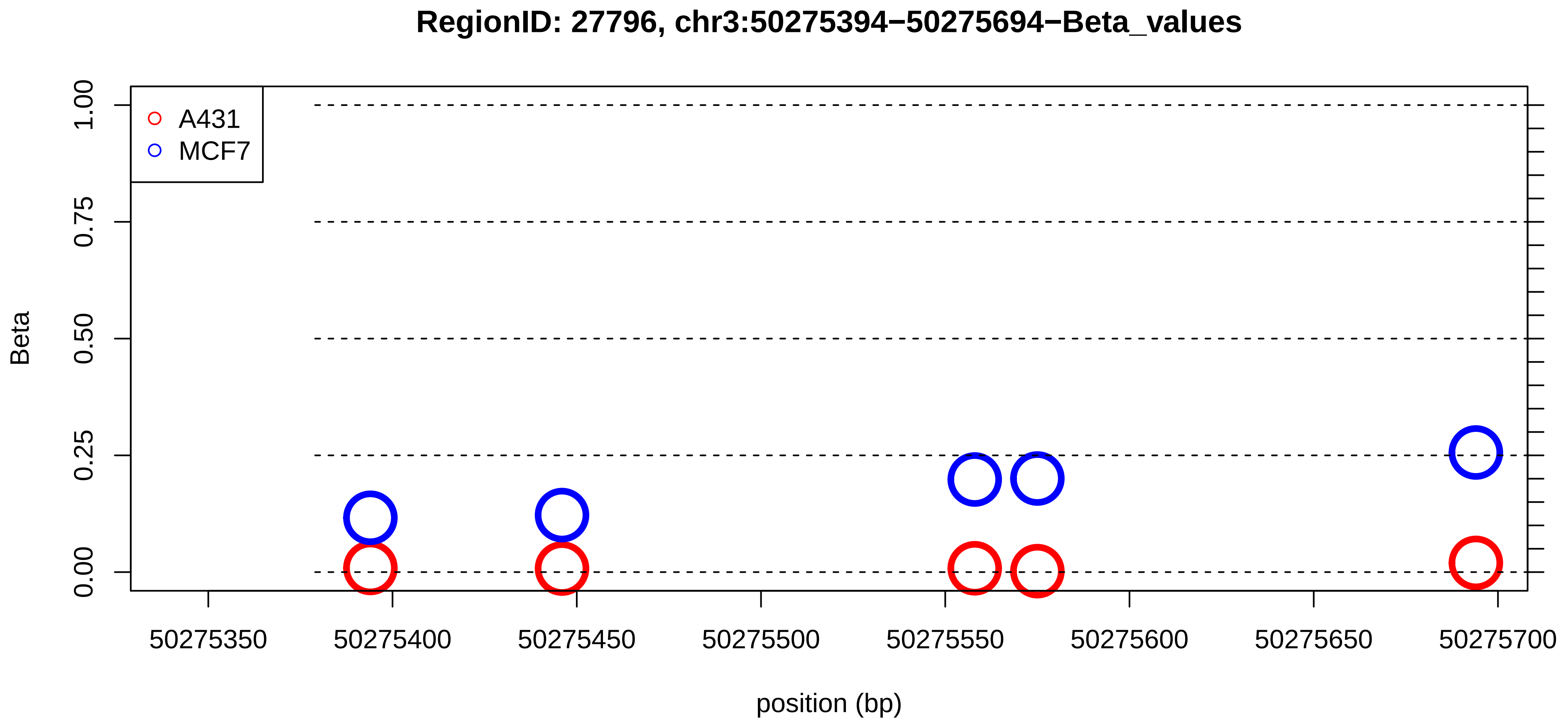
RegionID: 27796, chr3:50275394−50275694−Beta_values
A431
MCF7
Beta
0.00
0.25
0.50
0.75
1.00
50275350
50275400
50275450
50275500
50275550
50275600
50275650
50275700
position (bp)

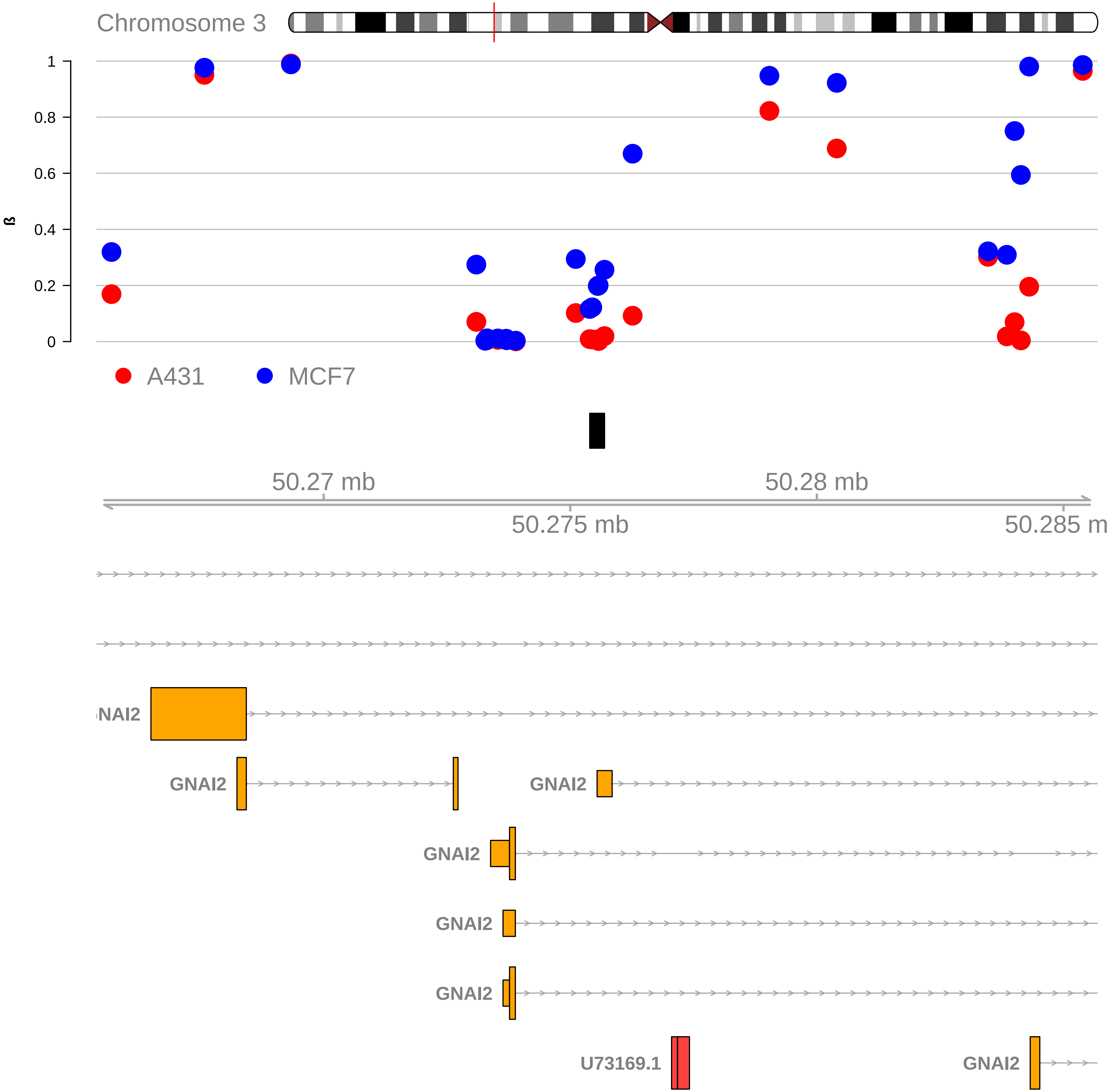

Chromosome 3
1
0.8
0.6
0.4
0.2
0
ß
A431
MCF7
50.27 mb
50.28 mb
50.275 mb
50.285 m
NAI2
GNAI2
GNAI2
GNAI2
GNAI2
GNAI2
U73169.1
GNAI2
